# Modulation of the vitamin D receptor by traditional Chinese medicines and bioactive compounds: potential therapeutic applications in VDR-dependent diseases

**DOI:** 10.3389/fphar.2024.1298181

**Published:** 2024-01-22

**Authors:** Minghe Yao, Patrick Kwabena Oduro, Ayomide M. Akintibu, Haifeng Yan

**Affiliations:** ^1^ Henan University of Chinese Medicine, Zhengzhou, China; ^2^ Collaborative Innovation Center of Research and Development on the Whole Industry Chain of Yu-Yao, Zhengzhou, China; ^3^ Jacobs School of Medicine and Biomedical Sciences, The State University of New York, University at Buffalo, Buffalo, NY, United States; ^4^ School of Community Health and Policy, Morgan State University, Baltimore, MD, United States; ^5^ The First Affiliated Hospital of Henan University of Chinese Medicine, Zhengzhou, China

**Keywords:** TCM, VDR, bioactive compounds, VDR-dependent diseases, 1α,25-dihydroxyvitamin D3

## Abstract

The Vitamin D receptor (VDR) is a crucial nuclear receptor that plays a vital role in various physiological functions. To a larger extent, the genomic effects of VDR maintain general wellbeing, and its modulation holds implications for multiple diseases. Current evidence regarding using vitamin D or its synthetic analogs to treat non-communicable diseases is insufficient, though observational studies suggest potential benefits. Traditional Chinese medicines (TCMs) and bioactive compounds derived from natural sources have garnered increasing attention. Interestingly, TCM formulae and TCM-derived bioactive compounds have shown promise in modulating VDR activities. This review explores the intriguing potential of TCM and bioactive compounds in modulating VDR activity. We first emphasize the latest information on the genetic expression, function, and structure of VDR, providing a comprehensive understanding of this crucial receptor. Following this, we review several TCM formulae and herbs known to influence VDR alongside the mechanisms underpinning their action. Similarly, we also discuss TCM-based bioactive compounds that target VDR, offering insights into their roles and modes of action.

## 1 Introduction

Traditional Chinese Medicine (TCM) has been an integral part of the healthcare system in China and other parts of the world for over three millennia. Recent studies on TCM herbal formulations and bioactive compounds have led to cutting-edge research findings, resulting in significant advances in medical knowledge about the therapeutic potential of TCM formulations. These studies have shown that TCM formulations can treat diseases and disorders like osteoporosis, cancer, alopecia, etc (An et al., 2016; Li et al., 2017; Zhang et al., 2022a). This is so because most of these TCM formulations have a rich variety of bioactive compounds, such as alkaloids, flavonoids, terpenoids, and polysaccharides, which have been demonstrated to modulate molecular cascades of biological activities, including anti-inflammatory, antioxidant, and immunomodulatory effects (Bai et al., 2019; Fang et al., 2020; Dai et al., 2021; Lu et al., 2021; Su et al., 2021; Cao et al., 2022a; Cao et al., 2022b; Gao et al., 2022). Importantly, recent emerging works show that some TCM-based compounds regulate Vitamin D receptor (*VDR*) [also known as 1α,25-dihydroxy vitamin D receptor or nuclear receptor subfamily 1 group 1 member 1 (NR1l1)] activity, potentially offering new opportunities for developing novel therapies for VDR-dependent diseases (Hou et al., 2017; Xiong et al., 2022).

*VDR* was first cloned and expressed in 1988 by Andrew Baker and his colleagues. It is part of the nuclear receptor 1 superfamily. VDR senses the active form of vitamin D3 (calcitriol or 1α,25-dihydroxy vitamin D3) (Baker et al., 1988) and, in response, triggers diverse specific gene expressions which are vital in the development and metabolism of an organism (Rochel et al., 2000). VDR can be found in the nucleus, cytosol, or plasma membrane, but in general, it is mainly localized to the nucleus (Tamura et al., 2017). *VDR* is conserved among many organisms, including dogs, frogs, rats, etc. In worms like *Caenorhabditis elegans*, the nuclear hormone receptor DAF-12 is the closest homolog to vertebrate VDR (Aghayeva et al., 2021). Sequence analysis of the *VDR* gene reveals highly conserved DNA-binding and hydrophobic ligand-binding domains, which are typical of nuclear receptors (Poon et al., 2012). *VDR* is found to be widely expressed in numerous tissues (Norman, 2008; Fagerberg et al., 2014; Carlberg and Muñoz, 2022). In addition, *VDR* is also found in some tumor tissues like mammary tumors, leukemia, etc. However, *VDR’s* expression and function are tissue-specific, and its native agonist autoregulates *VDR* expression levels *in-vivo* and in cells (Zella et al., 2006).

VDR is linked to many non-communicable diseases like osteoporosis, alopecia, diabetes, heart disease, and cancer. Activation of VDR by its ligand initiates signaling through VDR-retinoid-X-receptor heterodimers, which later bind to specific vitamin D response elements on DNA, resulting in the transcription of target genes. Today, our understanding of the functions of VDR continues to evolve. VDR drives the expression of genes that participate in cellular homeostasis, calcium and phosphate regulation, bone formation, cell growth and differentiation, immunity, and many more (Pike and Meyer, 2010; Christakos et al., 2016). Consequently, VDR represents an attractive drug target for many cellular metabolic pathways. Therefore, efforts are underway to unlock the tremendous value of TCM formulations and compounds as regulators of VDR activity in many diseases (Antony et al., 2010; Matsuo et al., 2013; Maestro et al., 2016; Sawada et al., 2018).

To provide a comprehensive overview of the potential of TCM as a modulator of VDR activity, this article will first establish a foundation by discussing VDR expression, functions, structure, and activation mechanism. We will then explore the therapeutic potential of VDR as a target for diseases resulting from VDR dysregulation and mutations. The main focus of this review will be on recent advances that demonstrate the potential of TCM-based formulations/compounds in modulating VDR.

## 2 Vitamin D receptor: expression, function, and structure

**FIGURE 1:**
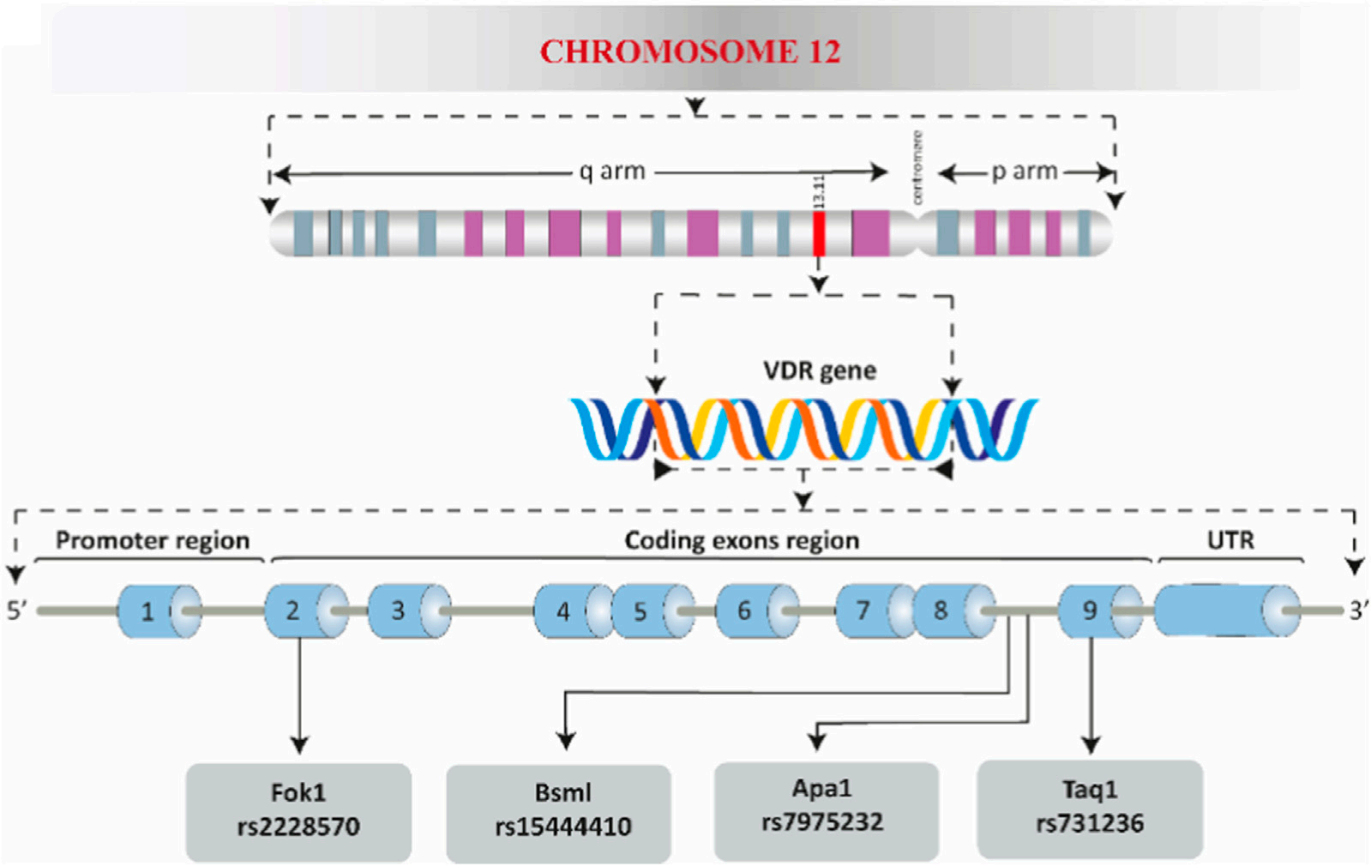
A schematic overview of the human VDR. The promoter region, coding exons region, the three prime untranslated region (UTR), and the well-known polymorphic sites are shown.

The *VDR* gene, which encodes vitamin D receptor protein, is located on chromosome 12q13.11 in humans (Figure 1) and chromosome 15 in mice (Haussler et al., 2013). It consists of 9 exons and has multiple isoforms (Esteban et al., 2005; Zenata and Vrzal, 2017). Various polymorphic sites have been identified in the *VDR* gene, including *VDR Apa1*, *Bsm1*, *Taq1*, *Fok1,* etc. These polymorphisms can affect mRNA expression patterns, protein translation efficiency, and VDR transcriptional activity (Fleet and Schoch, 2010; Han et al., 2017; Li et al., 2021). VDR expression has been detected in various cell types, including those involved in mineral homeostasis and immune cells (da Silva Teixeira et al., 2020; Provvedini et al., 1983; Kadowaki and Norman, 1984). The regulation of VDR expression is influenced by factors such as the presence of natural agonist, cellular molecules, including parathyroid hormone and retinoic acid hormone, and tissue-specific regulatory sites (Crofts et al., 1998; Lee et al., 2015).

**FIGURE 2:**
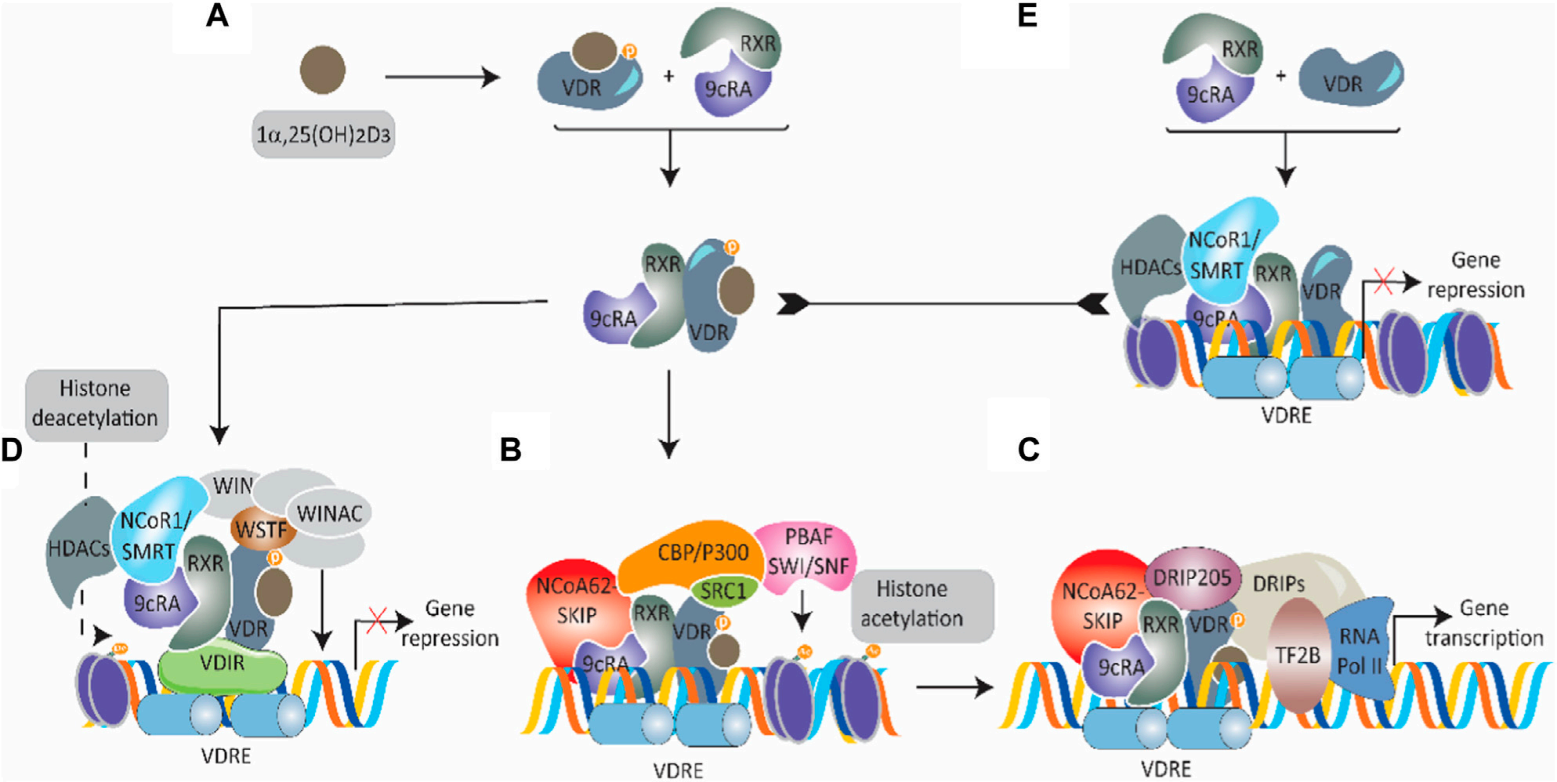
Overview of VDR transcriptional signaling. **(A)** A schematic representation of 1α,25-dihydroxy vitamin D3 [1α, 25 (OH)_2_D_3_]-induced activation of cytosolic VDR. On binding, VDR complexes with the retinoid-X-receptor coupled with 9-cis-retinoic acid (9cRA) and moves into the nucleus. **(B)** In the nucleus, the 1α, 25 (OH)_2_D_3_-VDR-RXR-9cRA complex binds to nuclear vitamin D response elements (nVDRE) and recruits several coactivators [nuclear coactivator—62 kDa-Ski-interacting protein (NCoA62-SKIP), steroid receptor coactivators (SRC1), CREB binding protein-p300 (CBP/p300), and SWI-2-related gene 1 associated factor (PBAF-SNF)] to promote histone acetylation and unwrap chromatin. **(C)** The mediator complex VDR-interacting proteins (DRIPs) are brought in by binding VDR-interacting protein 205. Transcription initiation is triggered by transcription factor 2B (TF2B) and RNA polymerase II. **(D)** In certain situations, transcriptional activation is prevented in 1α, 25 (OH)_2_D_3_ presence. This happens when 1α, 25 (OH)_2_D_3_-VDR-RXR-9cRA complex binds to a VDR-interacting repressor (VDIR) recognized by nVDRE, resulting in the dissociation of histone acetyltransferase (HAT) coactivators and the association of corepressors NCoR1/SMRT with histone deacetylase (HDAC) activity. The subsequent recruitment and interaction with the Williams syndrome transcription factor (WSTF) hinder gene transcription expression via the multifunctional ATP-dependent chromatin-remodeling complex (WINAC). **(E)** VDR that is not bound to 1α, 25 (OH)_2_D_3_ and is linked to RXR-9cRA moves into the nucleus and then binds to nVDRE. With the help of corepressors (NCoR1/SMRT) with histone deacetylase activity, transcription is inhibited.

The functions and molecular activation of VDR have been extensively studied since the cloning of the full-length VDR in 1988 (Baker et al., 1988). VDR’s function at the cellular level is primarily dependent on ligand binding, with 1α,25-dihydroxy vitamin D3 being a key agonist. The binding of an agonist to VDR initiates a sequence of events, starting with the formation of a heterodimer with the retinoid-X-receptor (Carlberg, 2004). This complex then translocates from the cytosol to the nucleus, although the specifics of this process remain unclear. In the nucleus, the ligand-bound VDR-retinoid-X-receptor complex interacts with vitamin D response elements to initiate transcription, aided by various coactivators (Prüfer et al., 2000). Without an agonist, VDR can still enter the nucleus but is associated with transcriptional corepressors that prevent gene transcription (Prüfer and Barsony, 2002; Carlberg, 2004; Deeb et al., 2007; Peleg and Nguyen, 2010; Toropainen et al., 2010; Takeyama and Kato, 2011) (Figure 2). The biological functions of VDR are diverse and complex, primarily due to its varied cell-specific actions. One of the most established roles of VDR is in the regulation of mineral metabolism and bone health, where it is known to suppress bone resorption (Nakamichi et al., 2017). Studies have shown that the absence of VDR leads to impaired bone growth, and VDR influences the expression of genes involved in bone differentiation and mineralization (Mathieu et al., 2001; van de Peppel and van Leeuwen, 2014). However, the relationship between VDR polymorphisms and bone diseases in humans remains uncertain, with conflicting results in clinical studies (Hunter et al., 2000; Thakkinstian et al., 2004; Uitterlinden et al., 2006). Besides bone health, VDR also plays a significant role in immune cell activation, cell proliferation, and differentiation, highlighting its broad functional spectrum in various cellular contexts (Bakke and Sun, 2018; von Essen et al., 2010; Halfon et al., 2015).

**FIGURE 3:**
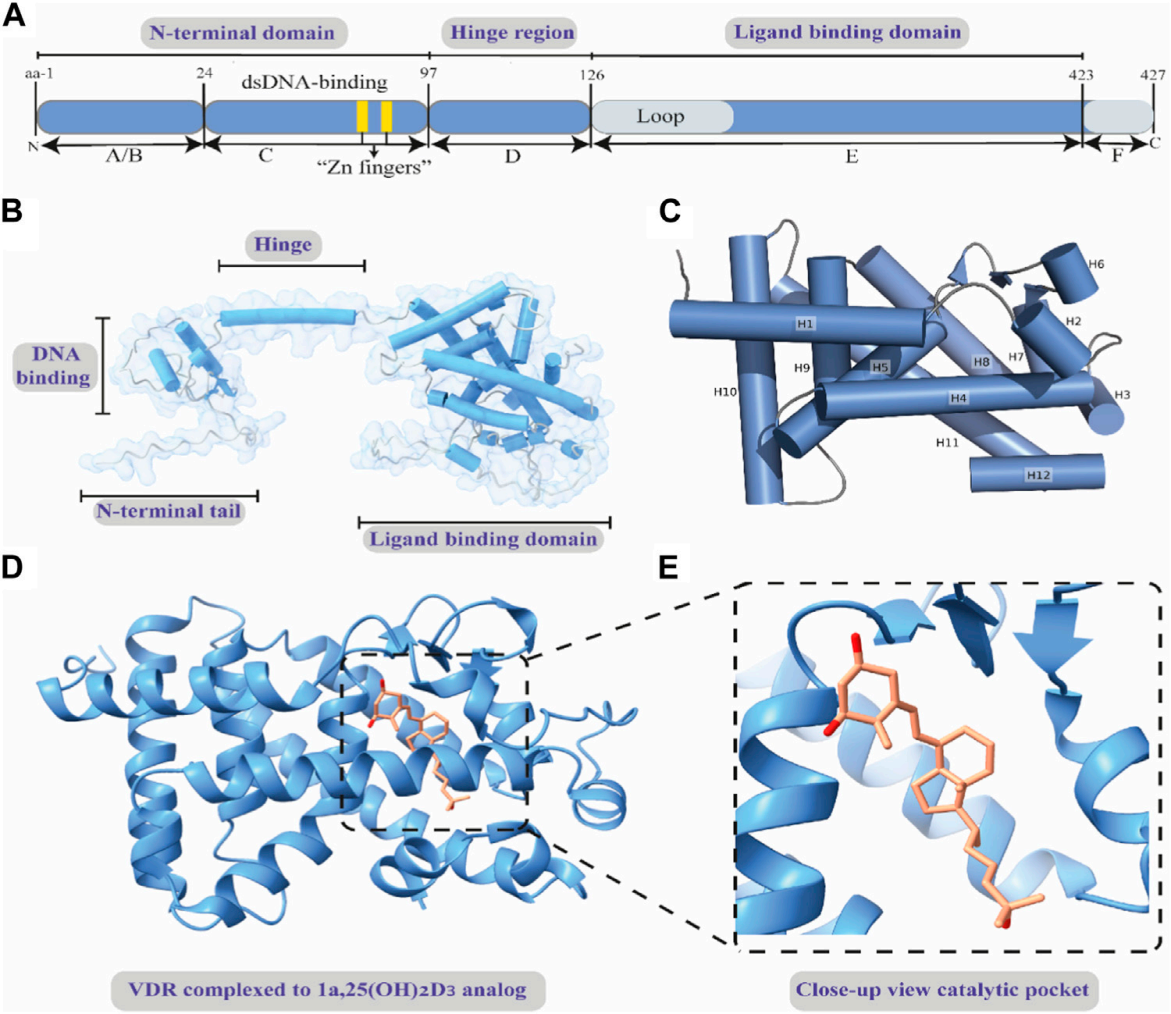
Basic structural features of human VDR. **(A)** Diagram of the functional domains of the human VDR. **(B)** The full-length 3-dimensional structure of apo-human VDR, showing the functional domains. **(C)** A close view of the crystal structure of the ligand-unbound ligand-binding domain of VDR. The α-helices are shown as cylindrical and numbered from H1-H12. **(D)** A cartoon representation of the ligand-binding domain of human VDR bound to 1α,25-dihydroxy vitamin D3 analog. **(E)** A magnified view of the binding site showing the binding mode of the inset ligand 1α,25-dihydroxy vitamin D3 analog. Full-length apo-human VDR (alpha fold model); Crystal structure of apo-human VDR ligand-binding domain (PDB ID: 3A78); and crystal structure of the nuclear receptor for vitamin D ligand binding domain bound to 1α,25-dihydroxy vitamin D3 analog (PDB ID: 1IE9).

The structural and biochemical features of VDR protein are multifaceted and crucial for its function. In human and mouse cells, the VDR consists of 427 and 423 amino acids, respectively, and is primarily located in the nucleus, although it can also be found in the cytoplasm and plasma membrane (Barsony and Prufer, 2002). VDR is characterized by its modular structure, which includes various domains such as A/B, C, D, E, and F (Figure 3), common to nuclear receptor 1 superfamily receptors (Wurtz et al., 1996; Rochel et al., 2000; Tocchini-Valentini et al., 2001). The A/B region at the N-terminus is relatively short, and the C domain is the highly conserved DNA binding domain featuring two “zinc finger” motifs. These motifs are critical for binding to DNA at vitamin D response elements, thereby facilitating VDR-induced transcription. The D domain, or flexible hinge, connects the DNA-binding and ligand-binding domains and plays a key role in nuclear translocation and in determining the orientation of the ligand-binding domain to DNA sequences (Michigami et al., 1999; Pike and Meyer, 2010; Nwachukwu and Nettles, 2012). The E domain is the ligand-binding portion, composed of ⍺-helices and β-sheets forming a hydrophobic pocket essential for binding ligands, coactivators, and corepressors (Mizwicki et al., 2007; Campbell et al., 2010; Wan et al., 2015; Sawada et al., 2018). This domain includes an activation function crucial for ligand-induced VDR activation and subsequent engagement with coactivators. The combined actions of the ligand-binding and DNA-binding domains influence VDR’s role in gene expression (Rochel et al., 2000; Orlov et al., 2012). Uniquely, VDR also features a F region, the function of which is not fully understood (Wurtz et al., 1996; Rochel et al., 2000; Tocchini-Valentini et al., 2001; Shaffer and Gewirth, 2004). Post-translational modifications, such as phosphorylation, SUMOylation, and potential ubiquitination, play significant roles in regulating VDR’s activity, localization, stability, and protein interactions (Arriagada et al., 2007; Zenata and Vrzal, 2017; Bakke et al., 2018). These modifications can also either enhance or suppress VDR’s transcriptional activities, exemplifying the complex regulation of VDR in cellular environments (Hsieh et al., 2004; Chi et al., 2009; Jena et al., 2012).

## 3 VDR and human diseases

**FIGURE 4:**
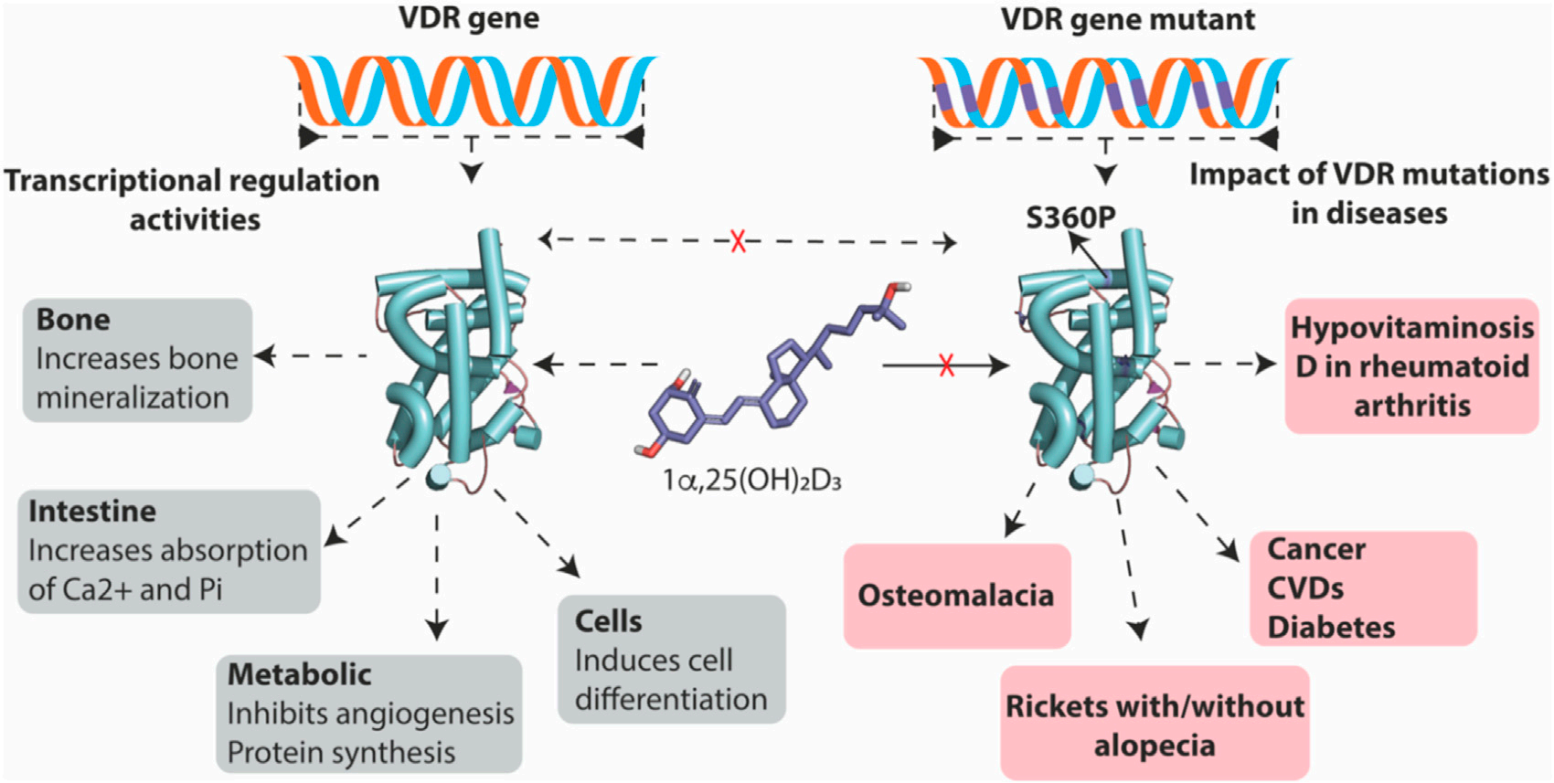
Disease mutants VDR but not wild-type VDR causes diseases. Wild-type VDR, upon binding to its ligand 1α,25-dihydroxy vitamin D3, plays a vital role in maintaining overall health by facilitating calcium and phosphate absorption and promoting bone mineralization. In addition, it also inhibits angiogenesis, promotes protein synthesis, and induces cell differentiation. In contrast, disease-associated mutants of VDR are resistant to 1α,25-dihydroxy vitamin D3, disrupting VDR signaling and leading to the development of various bone-related diseases and the progression of cancer, diabetes, and CVDs.

In recent decades, the therapeutic potential of VDR signaling in various diseases has been recognized (Figure 4). Modulating the VDR protein through genetic or drug-based strategies has improved our understanding of the many cellular processes controlled by VDR and how changes in VDR function can contribute to disease development or regression. VDR expression is present in most human body systems, and approximately 50–60 mutations in the VDR gene have been identified, most of which are found in the DNA-binding and ligand-binding domains, as well as other essential regions of the gene. These mutations have clinical implications and have been associated with severe hypocalcemic rickets, a condition linked to structural defects in bones in children (Hughes et al., 1988). One example of a VDR mutation is Gln152Xaa, which is found in the hinge region of the gene. This region is believed to be involved in the nuclear transportation of VDR or in supporting its association with specific response elements on DNA. The Gln152Xaa mutation is known to cause severe rickets associated with alopecia. This likely occurs because the mutation prevents VDR from carrying out its transcriptional activity to induce genes that control calcium and phosphate metabolism and hair growth signals. As a result, the body’s ability to maintain proper bone mineralization and hair growth signal is disrupted, leading to the development of severe rickets and total alopecia. Depending on the location of the mutations in the *VDR* gene, individuals with VDR mutation-induced rickets can be sub-grouped as having defects in hormone and DNA binding activity on VDR or restrains that prevent VDR nuclear localization. Most patients with VDR-dependent rickets condition have a range of signs and symptoms, including alopecia, hypocalcemia, hypophosphatemia, impaired bone formation, growth retardation, female infertility, uterine hypoplasia, impaired folliculogenesis, and hyperparathyroidism.

Another class of mutations of importance to our discussion here is missense mutations, point mutations in which a single nucleotide base change results in a codon that encodes for a different amino acid. Most missense mutations found in the *VDR* gene are associated with the rare disorder termed hereditary 1,25-dihydroxyvitamin D-resistant rickets or vitamin D-dependent rickets type IIA. It is worth noting that this type of rickets is different from vitamin D-dependent rickets type 1A—a disease caused by mutants in 25-hydroxyvitamin D3 1α-hydroxylase, the enzyme that catalyzes the synthesis of the active form (1α,25-dihydroxy vitamin D3) of vitamin D (Kitanaka et al., 1998). The first discovery of a missense mutation in the *VDR* gene was reported first in 1991 in Japanese rickets patients from heterozygous carrier parents (Saijo et al., 1991). Although it was not substantiated clearly on the exact missense mutant that was responsible for the vitamin D-dependent rickets type IIA, the authors’ analysis of the *VDR* gene from these patients strongly pinpointed that Arg47, located between the two zinc fingers, homozygous substitution is responsible for this disorder. However, subsequent advances in *VDR* gene analysis have confirmed this homozygous substitution and also further revealed unique homozygous missense substitutions such as Ser360Pro, Val346Met, Arg274Leu, and His305Gln in *VDR* gene in patients with vitamin D-dependent rickets type II (Tamura et al., 2017). In most cases, VDR transcriptional activity and ligand-binding affinity are lost entirely or partially impaired in all these missense mutants. For this reason, vitamin D-dependent rickets type II is resistant to 1α,25-dihydroxy vitamin D3 and its synthetic analogs treatment.

Due to space issues, we summarize three important VDR-dependent non-communicable diseases (Cancer, cardiometabolic disorders, and bone diseases) that are particularly important for our subsequent session discussion below.

### 3.1 Cancer and VDR

Cancer is one of the most therapeutically challenging diseases with extreme financial burdens on afflicted individuals. The link between VDR and cancer emanated from the “inconclusive” antineoplastic effects of the natural substrate 1α,25-dihydroxy vitamin D3. This led to the hypothesis that the *VDR* gene may act to repress tumorigenesis. It has also been observed that VDR is either highly expressed or downregulated in many histological types of cancers. For instance, in colon cancer, *VDR* mRNA is decreased (Zhang et al., 2020), while the opposite is true for basal cell carcinoma, squamous cell carcinoma, and breast, cervical, and ovarian cancers, suggesting a tissue-specific effect of VDR signaling. Of interest, although it remains unclear what drives the overexpression of VDR, the importance of overexpression of VDR in specific tumor types has been associated with better prognosis and prolonged survival in study participants. In fact, it is suggested that tumors with overexpression of VDR are more responsive to vitamin D supplementation. On the other hand, the low expression of VDR in host with colon cancers have been linked to colon cancer disease development and progression and a slight improvement in relapse-free survival at 5 years after vitamin D supplementation. Besides the tumor tissue-specific levels of VDR, several non-synonymous and synonymous single-nucleotide polymorphisms (*Fok1*, *Bsml*, *Apa1*, *Tru91*, and *Taq1*) in the *VDR* gene have been found. Most of them have been reported to be associated with either increased or decreased susceptibility to primary cancer and invasive and metastatic cancers of the prostate, breast, and head and neck. Also, data are available for their correlation with modifiable and unmodifiable risk factors of cancers. However, these associations are often based on small-sample sizes and do not account for ethnic differences and subgroup variations among study participants. Thus, light has not been adequately shed on the functional role of VDR signaling in cancers.

However, basic studies have tried to answer some of the questions relating to the role of VDR signaling in cancers by showing that VDR knockout results in increased susceptibility to chemicals-induced carcinogenesis. Thus, VDR signaling can potentially prevent tumorigenesis (Zinser et al., 2002; Zinser and Welsh, 2004; Zinser et al., 2005). For instance, hyperproliferation and enhanced mitotic activity in the descending colon were seen in laboratory-adapted *Vdr* ^−/−^ mice. To further examine how intestinal VDR contributes to the barrier function and protects against carcinogenesis, Zhang et al. utilized azoxymethane/dextran sulfate sodium-induced cancer model in intestinal VDR conditional knockout (VDR^∆IEC^) mice, 2D and 3D cell cultures, and human colon cancer samples. In a snapshot, the authors found that intestinal VDR helps keep the intestinal barrier structure and function and also acts as a tumor suppressor by regulating the JAK/STAT pathway in the intestine (Zhang et al., 2020). Another recent elegant study, which was aimed at investigating how VDR coordinates with other intestinal barrier structural integrity players like tight junctions to protect the host from colorectal cancer, found that intestinal epithelial VDR regulates cellular levels of tight junction protein claudin-5 to prevent inflammation and carcinogenesis in the colon. In addition, *CLDN-5* gene was identified as a novel target of VDR. This is because when the *VDR* gene was deleted, a decrease in claudin-5 and an increase in tumor development were observed. In contrast, increased claudin-5 and protection against tumorigenesis in intestinal epithelial cells were found after enhancing VDR activity (Zhang et al., 2022b). In sum, VDR status and signaling may be related to cancer prevention or retrogression. However, many critical translational questions must be explored and answered on the cellular and molecular mechanisms by which VDR prevents the survival of cancer cells and inhibit angiogenesis.

### 3.2 Cardiometabolic diseases and VDR

Cardiovascular diseases and metabolic disorders, collectively known as cardiometabolic diseases, remain a leading cause of disease-related mortality worldwide. In an effort to understand the factors that contribute to the development and progression of these conditions, researchers have been studying the role of metabolic genes in cardiometabolic diseases. One area of interest is the link between cardiometabolic events and the seasons, as several observational studies have shown that cardiometabolic diseases tend to be more common during the winter months (Motiwala and Wang, 2012). These observations have spurred researchers to investigate the role of VDR signaling in cardiometabolic diseases such as diabetes, hypertension, and metabolic syndrome. A growing body of research using human and animal disease model samples has shown that VDR is a metabolic gene and that individuals with metabolic syndrome and vascular diseases tend to have downregulated VDR expression. This suggests that VDR may have an essential role in the development and progression of cardiometabolic diseases and that targeting VDR signaling may be a promising approach for preventing and treating these conditions (de Albuquerque Borborema et al., 2021). In support of this idea, it has been discovered that VDR plays a key role in regulating cellular processes that are essential to cardiovascular and metabolic health, such as cell proliferation and differentiation, metabolic homeostasis, tissue mineralization, protein synthesis and degradation, and ion transport for catalysis. In fact, most cardiovascular and metabolic cell types, including endothelial cells, adipocytes, hepatocytes, smooth muscle cells, immune cells, cardiomyocytes, and platelets, have been found to express VDR strongly (Somjen et al., 2005; Tishkoff et al., 2008).

Secondary bile acids, which are derived from gut microbial metabolism, are VDR ligands and regulators of VDR expression. Multiple gut microbiome analyses using 16s rRNA gene sequencing have shown that alterations in the gut microbiome composition can lead to cardiovascular and metabolic diseases. In fact, a genome-wide association and shotgun metagenomic analysis published in *Nature Genetics* found that the gut microbiome composition of *VDR* ^−/−^ mice differed significantly from that of control mice that express VDR. Additionally, VDR-mediated signaling was found to be critical in the gut-liver signaling axis and microbial co-metabolism (Wang et al., 2016), suggesting that VDR signaling can influence gene targets that are relevant to cardiometabolic health and that changes in *VDR* gene expression can lead to disruptions in the gut microbiome that can cause cardiometabolic diseases. Furthermore, several observational studies have suggested that *VDR* gene polymorphic alleles and genotypes are associated with an increased risk of cardiovascular and metabolic diseases in certain populations, such as pregnant women, offspring, and Asians (Apaydın et al., 2019; Mokhtar et al., 2019). For example, it has been found that individuals carrying the *VDR* SNPs *Apa1*, *Taq1*, and *Bsm1* genotypes were more likely to be obese, while those with the *Fok1* genotype were at an increased risk of heart failure and hypertension (Abouzid et al., 2021). These findings highlight the potential role of VDR in the development of cardiometabolic diseases and the need for further research to understand the mechanisms underlying this relationship.

Although this mini-review in its entirety seeks to focus on VDR, it is worth noting that several experimental pieces of evidence have shown that exposure 1α,25-dihydroxy vitamin D3 in cardiovascular and metabolic cell types, leads to a decrease in endothelial inflammation, improves flow-mediated dilation, reduces endothelium-dependent contraction, enhances nitric oxide release, improves tissue mineralization, decreases cholesterol uptake and foam cell formation, modulates immune cells differentiation, etc. (Norman and Powell, 2014). Besides, significant associations between low vitamin D intake and adverse endpoints of cardioembolic diseases have been found. It has been suggested that adequate supplementation with vitamin D or 1α,25-dihydroxy vitamin D3 has favorable outcomes on biochemical and metabolic parameters (Breslavsky et al., 2013), suggesting a therapeutic implication for VDR signaling. However, despite the asserted beneficial effect of VDR signaling on the cardiovascular system, other epidemiological studies, mainly priming VDR signaling via vitamin D supplementation, suggest a disease-causal role for VDR in the pathogenesis of cardiovascular and metabolic diseases (Kayaniyil et al., 2010). These deleterious effects of VDR signaling, perhaps, can be attributed to the high intake of vitamin D used in most of these studies. Altogether, the role of VDR signaling cannot be overlooked in cardiometabolic health, but several knowledge gaps, like the tissue-specific pattern effects of VDR and its relevance in a specific cardiometabolic disease, need to be addressed.

### 3.3 Bone disorders and VDR

Bone is widely recognized as the primary target of vitamin D, as numerous studies have shown that 1α,25-dihydroxy vitamin D3 supplementation can alleviate bone diseases such as rickets, osteomalacia, osteosclerosis, and osteoporosis. Osteoblasts, osteoclasts, chondrocytes, and osteocytes are important human bone cells that regulate bone formation, resorption, and mineralization. These cells express the *VDR* gene, and mice that lack this gene develop severe rickets and osteomalacia. These findings demonstrate the crucial role of the VDR-1α,25-dihydroxy vitamin D3 complex in maintaining healthy bones (Nakamichi et al., 2018). Besides, it is well established that VDR is the epicenter regulator of bone formation, resorption, mineralization, and metabolism. Mainly via the expression of genes that interact with other key cellular factors and other metabolic pathways that help to ensure the proper development and mineralization of bones (Luo et al., 2018). However, despite the known role of the VDR-1α,25-dihydroxy vitamin D3 complex in regulating bone mineralization, formation, and resorption, our understanding of these processes is still incomplete. This is because studies using *VDR* ^*−/−*^ genetic systems to investigate the functions of VDR in bone-specific cells have yielded conflicting results. For example, some studies using osteoblast lineage-specific *VDR cKO* mice and osteocyte-specific *VDR cKO* mice have reported significant abnormalities in bone mineralization and disruptions in bone formation and resorption processes in these mice, while others have found the opposite (Verlinden et al., 2019). The conflicting results of these studies may be due to differences in the specificity of the cell systems used to knockout *VDR* or the effectiveness of the Cre activity in the Cre transgenic mice that were often used to delete *VDR* gene. Therefore, further research is needed to clarify the role of VDR in regulating bone mineralization, formation, and resorption (Verlinden et al., 2019).

In elderly individuals, the expression of VDR and its target genes, which are crucial for cell differentiation, is decreased. This decrease in VDR expression is believed to be caused by well-known risk factors for diseases such as aging. As a result, the ability of the VDR-1α,25-dihydroxy vitamin D3 complex to regulate bone health may be impaired in the elderly, contributing to the increased risk of bone-related diseases (Jiang et al., 2020). This is consistent with the well-established idea that aging makes us more susceptible to diseases, which explains why older adults are at increased risk of developing metabolic bone diseases such as osteoporosis, rheumatoid arthritis, and osteomalacia. Notably, studies involving elderly patients have found that vitamin D intake can promote longevity by inducing VDR expression and enhancing metabolic bone processes that have declined due to aging. As with other diseases discussed earlier, the effect of VDR polymorphisms on the risk of bone diseases in specific ethnic populations has also been studied. A meta-analysis of almost 10,000 people recently found that *ApaI*, *BsmI*, and *FokI* polymorphisms, but not *TaqI* polymorphism, were associated with the metabolic bone disease osteoporosis. Further subgroup analysis focused on ethnicity revealed that *ApaI*, *BsmI*, and *TaqI* polymorphisms were significantly associated with the incidence of osteoporosis in Caucasians, while VDR polymorphisms *BsmI* and *FokI* were linked to an increased risk of osteoporosis among Asians. These findings highlight the importance of considering the role of VDR polymorphisms in the development of bone-related diseases in different ethnic populations (Jiang et al., 2022). In summary, while the exact mechanisms by which VDR-mediated transcription promotes osteogenesis and osteoblastic bone formation while also suppressing osteocyte senescence and osteoclastic bone resorption remain unclear, the significant role of VDR in bone formation and bone-related diseases is undeniable. Further research is needed to better understand the specific mechanisms by which VDR regulates these processes and to identify potential therapeutic strategies for maintaining healthy bones and treating bone-related diseases.

### 3.4 Kidney diseases and VDR

Kidney diseases, encompassing a range of pathologies such as IgA nephropathy, idiopathic nephrotic syndrome, renal cell carcinoma, diabetic nephropathy, and lupus nephritis, pose serious health challenges globally. Recent strides in understanding the role of the VDR in these diseases offer a promising therapeutic frontier. VDR activation, shown to protect renal function, operates through a multitude of mechanisms: it suppresses the renin-angiotensin system, reduces inflammation, inhibits fibrogenesis, enhances mitochondrial function, attenuates autoimmunity, and reduces renal cell apoptosis (Yang et al., 2018). Such multifaceted roles underscore VDR’s potential as a therapeutic target. For example, in the context of lupus nephritis, a notable condition marked by increased disease activity and severity, a correlation has been observed with decreased VDR expression in renal tissues (Huang et al., 2021). This finding is pivotal as it suggests that VDR not only plays a role in disease manifestation but also in its progression. VDR agonists have emerged as a beacon of hope in these scenarios, showing efficacy in reducing renal inflammation and restraining the nuclear translocation of inflammatory mediators (Sanchez-Niño et al., 2012).

Another particularly intriguing study in the realm of IgA nephropathy revealed differential expressions of VDR and the calcitriol-synthesizing enzyme, 1alpha-OHase (Arapović et al., 2021). Patients showed elevated VDR expression in distal tubular cells, contrasted with a marked decrease in 1alpha-OHase (Arapović et al., 2021). This disparity opens new avenues for understanding the disease’s molecular underpinnings and, possibly, its management. The role of VDR extends into the broader spectrum of chronic kidney disease, where its diminished levels and activity exacerbate conditions like secondary hyperparathyroidism and renal fibrosis (Bakdash et al., 2014; Barragan et al., 2015). This insight gains practical significance in experimental models of chronic renal failure (CRF), where VDR agonists like paricalcitol and calcitriol have demonstrated remarkable anti-fibrotic effects, offering a therapeutic window (Martínez-Arias et al., 2021). Moreover, the genetic landscape of VDR in kidney diseases has been illuminated by a comprehensive meta-analysis. This analysis identified specific VDR gene polymorphisms, such as *BsmI, FokI, and TaqI*, associated with an increased risk of renal diseases, including end-stage renal disease (Xu et al., 2022). These genetic insights not only deepen our understanding of the disease etiology but also pave the way for personalized medicine approaches in renal pathology.

The accumulating evidence highlights a significant yet complex relationship between VDR and kidney diseases. It urges the scientific community to delve deeper into this nexus, exploring how VDR modulation can be effectively harnessed in therapeutic strategies. This endeavor demands a multi-dimensional approach, integrating molecular biology, genetics, and clinical research to unravel the connection of VDR in renal health and disease. The ultimate goal is to translate these findings into clinically effective, patient-centric therapies, offering new hope in the battle against kidney diseases. As such, the path ahead is laden with opportunities for groundbreaking research and innovative therapeutic developments, beckoning researchers to explore this promising and largely uncharted research field.

## 4 Therapeutic applications of TCM and bioactive compounds in VDR-dependent cancer diseases

TCM has a history spanning over two thousand years, offering a unique perspective on health, disease, and healing that differs from Western medicine. TCM embraces a holistic approach, viewing the body as an interconnected system rather than a collection of individual parts. Its principles are rooted in the concept of balance and harmony, emphasizing the importance of the body’s ability to self-regulate and heal (Zou, 2016). In recent years, the relationship between TCM and modern science has garnered significant attention. On a yearly basis, several scientific investigations come out to demonstrate that, indeed, TCM formulae, herbs, and bioactive compounds exert their therapeutic outcomes by targeting several regulating several diseased biomolecules, hence improving health and alleviating disease (Liu et al., 2014; Zhou et al., 2021). Similarly, studies have revealed that TCM formulae, herbs, and compounds also possess the ability to influence the activity of the VDR (Kuai et al., 2020; Chen et al., 2021; Xiong et al., 2022). The discovery of VDR-modulating properties in TCM formulae components, herbs, and TCM compounds opens up new avenues to uncover novel therapeutic approaches for VDR-dependent human diseases, potentially offering safer and more effective treatment options.

### 4.1 VDR-modulating traditional Chinese medicine formulae and herbs: examples and mechanisms

TCM, with its diverse array of formulas and herbs, has been discovered to interact with and modulate VDR activity in ways that are crucial for the alleviation or improvement of VDR-dependent diseases (Xiong et al., 2022). TCM formulas and herbs have been used in numerous clinics to treat, for example, bone and skin disorders (Si et al., 2018; Wu et al., 2018; Wang et al., 2020). In our world today, particularly in the aging population, for instance, TCM has emerged as an economical alternative to well-known western medicines for bone discomfort and aging skin. This is so because, in various VDR-dependent diseases such as psoriasis, osteomalacia, joint sprains, osteoporosis, inflammatory disorders, and even broken bones, topical applications of TCM formulas or herbal remedies have proven beneficial (Li et al., 2017; Zhang et al., 2022a). For example, clinical studies using TCM for the management of musculoskeletal disorders, particularly bone fractures, osteoporosis, and rheumatoid arthritis, have reported some significant success (Li et al., 2007; He et al., 2008; Xu et al., 2023). In addition, it has been shown that some TCM natural products investigated in laboratory settings for the treatment of osteoporosis appear to have both anabolic and anticatabolic effects, enhancing osteogenesis and reducing unbalanced osteoclast activity, resulting in improved bone mineral density and biomechanical properties, and reduced bone microstructural degeneration (An et al., 2016). These impressive findings suggest that TCM formulas and herbs act at the molecular level to influence, for example, osteoporosis-dependent genes, particularly VDR, to promote these beneficial effects. As seen in Table 1, a comprehensive list of TCM formulas and herbs have been studied for their ability to modulate VDR activity. These TCM interventions range from single herbs to complex formulas, each providing unique insights into how VDR activity can be influenced to alleviate VDR-dependent diseases such as osteoporosis, cancer, psoriasis, diabetes, etc.

**TABLE 1:** Traditional Chinese medicine formulas and herbs target VDR activity to improve VDR-dependent diseases.

Traditional Chinese medicine formula/Herb	Name	VDR-dependent mechanism	VDR-dependent disease	Refs
Traditional Chinese medicine formulae	Fuzheng Xiaojijinzhan (*扶正消积金盏*)	Upregulate the expression levels of VDR and E-cadherin, downregulate the expression levels of TGF-*β* and Snail1, regulate the VDR/TGF-β/Snail1 signaling pathway	Colorectal cancer liver metastasis	Li et al. (2022a)
	Bu-Shen-Jian-Pi-Yi-Qi Therapy (*补肾健脾益气法*)	Increase BMD and BMC, improve biomechanical properties, elevate VDR mRNA level, and enhance response sensitivity of 1, 25(OH)2D_3_, and reduce S-Ca/P	Alcohol-induced osteoporosis	Ren et al. (2016)
	HuanglianGanjiang Tang (*黄连干姜汤*)	Increased the expression of VDR and caspase 8, which can reduce the phosphorylation of RIPK1 and RIPK3	Ulcerative colitis	Xiong et al. (2022)
	Jiangzhi granule (*降脂颗粒*)	Activate/Upregulate of the endogenous VDR	Non-alcoholic steatohepatitis	Cao et al. (2022b)
	Psoriasis 1 (*银屑病1号*)	Decrease the inflammation mediated by T lymphocytes with psoriasis through inhibiting VDR-mediated STAT4 inactivation	Psoriasis	Gao et al. (2020)
	Psoriasis 1 (*银屑病1号*)	Reduce psoriasis-like skin inflammation by inhibiting the VDR-mediated nuclear NF-*κ*B and STAT signaling pathways	Psoriasis	Sun et al. (2018)
	Zhuanggu Guanjie Pill (*壮骨关节丸*)	Increase the expression of VDR and CAR in female rats, induce the CYP1A2 and CYP2B6 activities of both male and female rats	Osteoporosis	Chai et al. (2021a)
	Migu capsule (*密骨胶囊*)	Enhanced bone mineral density, raise calf muscle/weight, and improve the expression of calf muscle VDR mRNA in the small intestine	Osteoporosis	Wang et al. (2009)
	Strengthening Spleen prescription (*健脾方*)	Remain bone mineral density and improve the expression of calf muscle VDR mRNA in the small intestine	Osteoporosis	Wang et al. (2009)
	Jueyin granules (*决银颗粒*)	Upregulate VDR expression and downregulate p-STAT3 expression	Psoriasis	Kuai et al. (2020)
Traditional Chinese medicine herb	*Astragalus Membranaceus* (*膜荚黄芪*)	Improve BMD and BMC, elevate the contents of calcium and phosphorus, increase the expression of Klotho, VDR, and CYP27B1, decrease the expression of FGF23 and CYP24A1	Osteoporosis	Chai et al. (2021b)
	*Fructus Ligustri Lucidi* (*女贞子*)	Upregulated the expression of VDR in the duodenum, preserve bone quality through regulation of the calcium balance and intestinal SCFAs production	Osteoporosis	Chen et al. (2021)
	*Fructus Ligustri Lucidi* (*女贞子*)	Regulate vitamin D metabolism in kidney, increase the expression of VDR in duodenum, and reduce the expression of CaSR in parathyroid gland and kidney	Type 1 diabetes	Sha et al. (2017)
	*Mori Folium* (*桑叶*)	Regulate the calcium and redox homeostasis via PTH/VDR/CaBP and AGEs/RAGE/Nox4/NF-κB signaling to eliminate oxidative stress (Upregulate the expression of VDR)	Diabetic osteoporosis	Liu et al. (2018a)
	*Patrinia villosa Juss*. (*白花败酱草*)	Impact bile acid levels, activate VDR, and inhibit the overactivation of NF-*κ*B signaling pathways	Ulcerative colitis	Wang et al. (2022)

## 5 Natural bioactive compounds targeting VDR activity

The active natural metabolite of vitamin D3, 1α,25-dihydroxy vitamin D3, binds to the VDR-ligand binding domain, initiating physiological responses linked to VDR stimulation. This interaction has been discovered to have numerous positive effects on health and disease. Therefore, scientists have been striving to create chemical alterations that can produce active analogs of 1α,25-dihydroxy vitamin D3 with the desired pharmacokinetic and pharmacodynamic profiles, as well as minimal toxicity. In the past, these endeavors were based on “trial and error” or “educated guess” methods, but this changed in 2000 when the first X-ray crystal structure of the VDR-ligand binding domain bound to 1α,25-dihydroxy vitamin D3 was deciphered. This significant breakthrough allowed for a more targeted and effective approach to the development of 1α,25-dihydroxy vitamin D3 analogs (Christakos et al., 2016). Consequently, the crystal structure of the VDR-1α,25-dihydroxy vitamin D3 complex sparked a revolution in the rational design of second-generation 1α,25-dihydroxy vitamin D3 analogs.

**FIGURE 5:**
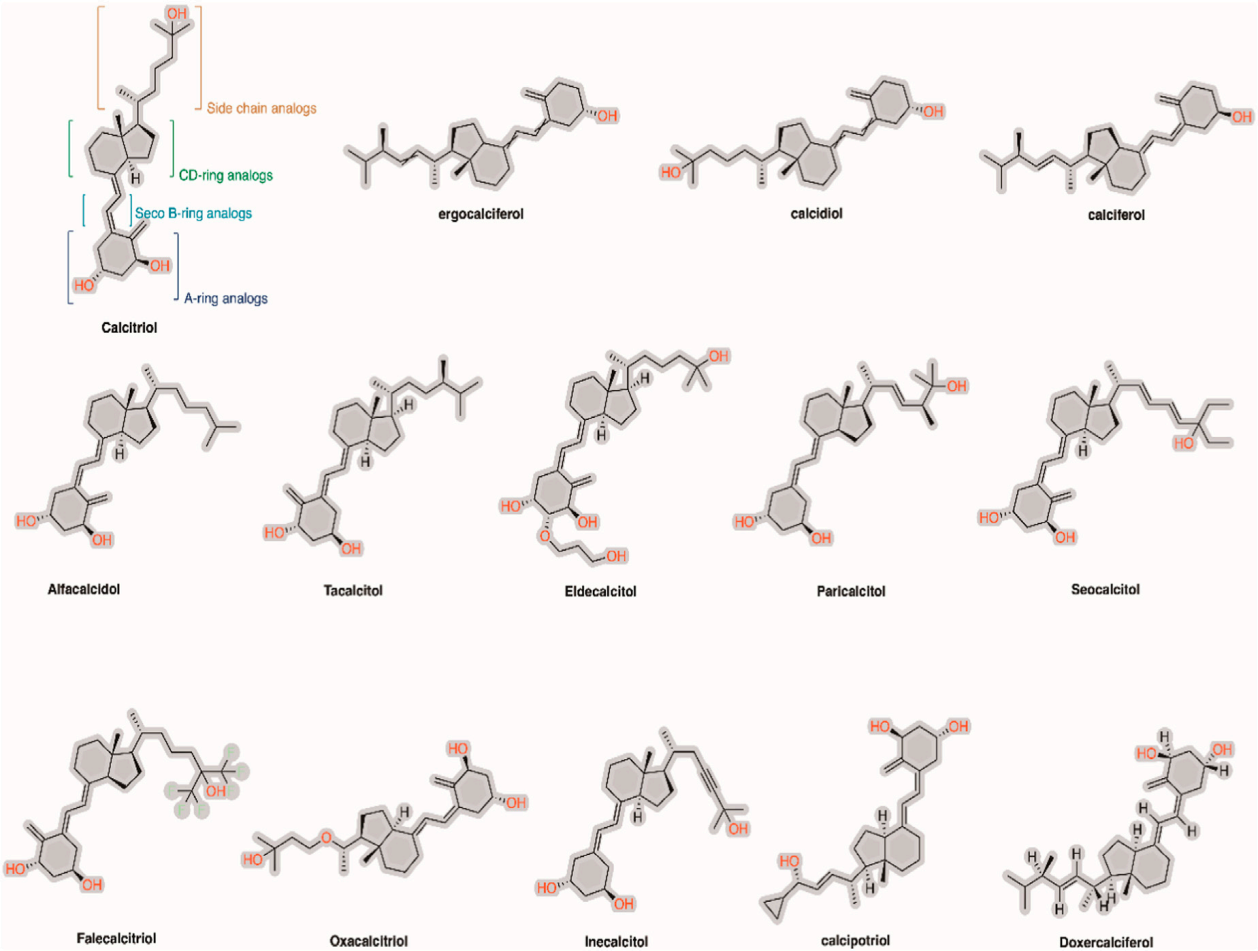
Chemical structure of 1α,25-dihydroxy vitamin D3 and its analogs on the market.

To date, approximately 5,000 synthetic or semi-synthetic 1α,25-dihydroxy vitamin D3 analogs have been developed by pharmaceutical and academic research institutions. These analogs can be classified based on the chemical modifications made to 1α,25-dihydroxy vitamin D3 structural motifs, including A-ring analogs, Seco B-ring analogs, CD-ring analogs, and side chain analogs (Figure 5). Most of these analogs have been demonstrated to have reduced calcemic side effects and potent therapeutic effects that are similar to or better than those of the parent 1α,25-dihydroxy vitamin D3 compound in cell cultures, animal models, and several human diseases. In addition to the steroidal VDR modulators, nonsteroidal VDR modulators, including LY2108491, LY2109866, and LG190119, have also been reported. Their efficacy in diseases is currently limited to studies using laboratory-adapted animal species (Maestro et al., 2019). However, it is expected that more nonsteroidal compounds capable of modulating the activity of VDR will be discovered in the future, potentially offering promising therapeutic prospects for VDR-dependent human diseases.

### 5.1 TCM-based bioactive compounds and VDR activity

TCM-based compounds refer to the bioactive molecules derived from medicinal herbs and other source formulations used in TCM-based clinics. These compounds encompass a broad range of chemical substances belonging to the phytochemicals, such as alkaloids, flavonoids, saponins, polysaccharides, and terpenoids, among others. Examples of TCM-based compounds include artemisinin, which is extracted from the plant *Artemisia annua* and is used as a potent antimalarial drug, and berberine, which is found in several plants used in TCM and has been shown to have antibacterial, anti-inflammatory, neuroprotective and anti-diabetic effects (Wang et al., 2017; Feng et al., 2020). Other examples include ginsenosides from ginseng, which have a variety of therapeutic effects, and curcumin from turmeric, known for its anti-inflammatory and antioxidant properties via the radical scavenging activity (Hung et al., 2021). The therapeutic potential of TCM-based bioactive compounds is vast and continually being explored. Their diverse chemical scaffolds and biological activities make them an excellent source of novel drugs or therapeutic agents against numerous human diseases. Because, these bioactive compounds have been found to possess various pharmacological properties, including modulating cellular proteins or enzymes activity at the cellular and molecular levels (Zhang et al., 2019; Yang et al., 2020).

As discussed earlier, dysregulation or alterations in VDR activity have been implicated in a variety of diseases, including osteoporosis, autoimmune disorders, and certain types of cancer. Therefore, targeting VDR activity has therapeutic potential in the treatment of VDR-dependent diseases. Recent research indicates that certain TCM-based compounds can interact with and modulate VDR activity, presenting a promising avenue for the treatment of VDR-dependent diseases. For instance, some TCM-based bioactive compounds might enhance *VDR* gene expression or antagonize VDR receptor, thereby positively or negatively influencing the biological processes regulated by VDR and potentially ameliorating the symptoms or progression of VDR-dependent diseases like bone diseases, neurodegenerative diseases, etc. (Ding et al., 2021; Luo et al., 2022). Moreover, considering that low vitamin D levels and VDR *Fokl* variants are linked to sepsis susceptibility (Yang et al., 2022), a study on the TCM-based bioactive compound emodin demonstrated that emodin treatment can increase both mRNA and protein expression of VDR and its downstream molecules. It can also inhibit the expressions of TNF-α, IL-6, and MDA in serum and tissue and increase the levels of SOD and GSH. This regulation of the VDR/Nrf2/HO-1 signaling pathway can improve immune function and protect against sepsis-induced intestinal barrier damage (Shang et al., 2021).

Below, we delve deeper into the intriguing world of TCM-based bioactive compounds and their impact on VDR activity. We explore how these bioactive substances, rooted in centuries of traditional use, can offer novel solutions to improve the outcomes of VDR-dependent diseases. This discussion sheds light on the untapped potential of TCM-based bioactive compounds in addressing complex metabolic health challenges through the lens of VDR signaling and activity.

**1. Astragalus polysaccharide:** The *Astragalus membranaceus* plant, also known as Huang Qi, belongs to the family Fabaceae and is native to China. The roots of *A. membranaceus* are used in TCM for their immune-modulating, cardiotonic, hepatoprotective, and diuretic properties (Liu et al., 2018b). Astragalus polysaccharide is a high molecular weight heteropolysaccharide compound isolated from the *A. membranaceus* plant, which is commonly used in TCM. This polysaccharide is made up of various monosaccharides, including glucose, galactose, and arabinose, among others. It is a member of the polysaccharide class of phytochemicals.

The pharmacological activity of astragalus polysaccharide has been extensively studied. It has been shown to have antioxidant, anti-inflammatory, antiviral, and immunomodulatory properties (Li et al., 2022b). Additionally, Astragalus polysaccharide has shown immense potential in the treatment of various diseases, including cancer, diabetes, cardiovascular diseases, and liver diseases (Li et al., 2022b). Its efficacy extends not only to alleviating these conditions but also to combating sepsis, as revealed by a recent study (Li et al., 2022c). This study suggested that the upregulation of adrenocortical VDR activity and vitamin D levels might be the mechanism by which astragalus polysaccharide improves sepsis outcomes.

Using a septic rat model, the authors found that treatment with astragalus polysaccharide led to an upregulation of the vitamin D metabolic axis. This upregulation had a dual effect: it suppressed the expression of inflammatory cytokines while promoting the production of adrenal cortical proteins such as VDR. The latter is especially significant as these proteins play a crucial role in modulating the gut microbiota flora, specifically, those species that produce short-chain fatty acids. These fatty acids can directly or indirectly mitigate sepsis processes and renal damage induced by sepsis (Li et al., 2022c). This is consistent with a previous study that also show the protective effects of astragalus polysaccharide on sepsis-indued acute kidney injury (Sun et al., 2021). Importantly, the significance of these findings are underscored by the established correlation between vitamin D levels and disease severity, including mortality (Parekh et al., 2017). Patients with vitamin D deficiency have a higher probability of developing sepsis and acute renal failure compared to healthy individuals (Olejarova et al., 2019). Thus, the potential of astragalus polysaccharide to upregulate vitamin D activity makes it a promising target for improving sepsis outcomes.

However, the path to developing astragalus polysaccharide into a therapeutic drug is not without its challenges. A critical hurdle lies in the standardization of the plant material, given the complex phytochemical mixture found in *A. membranaceus* (Fu et al., 2014). Furthermore, the current lack of well-designed human clinical trials evaluating the efficacy and safety of astragalus polysaccharide presents another significant obstacle. Despite these challenges, promising results from preclinical studies advocate for more comprehensive human-based studies. This further investigation is vital to truly unlock and harness the full therapeutic potential of astragalus polysaccharide.

**2. Bufalin**: Is a cardiotonic digoxin-like steroid that is extracted from the skin and parotid venom glands of the Chinese toad species *Bufo melanostictus* and *Bufo gargarizans*, also known as Chan Su. This TCM material has been used for centuries as a painkiller and for the treatment of heart failure, cancer, and other diseases (Long et al., 2022). Bufalin is one of the major active components of Chan Su, alongside other bufadienolides such as bufalon, resibufagin, gambufotalin, bufataline, and cinobufotalin (Wei et al., 2019). Bufalin has a chemical structure consisting of a five-member lactone ring with a C-24 steroid backbone and four fused rings. It is a member of the cardiotonic steroid class of phytochemicals. Bufalin’s pharmacological activities have been widely studied, including anti-inflammatory, anti-angiogenic, and anti-viral activities, as well as effects on the cardiovascular and nervous systems (Tsai et al., 2012; Wei et al., 2019). Most importantly, bufalin has shown therapeutic potential in the treatment of various types of cancers, including liver cancer, pancreatic cancer, and breast cancer, because of its ability to act at the molecular level to arrest cell proliferation and induce several forms of cell death, including apoptosis and necroptosis (Soumoy et al., 2022).

Clinical studies have demonstrated that a deficiency in vitamin D is connected to an elevated risk of cardiovascular disease and cancer (see the above discussion). Therefore, vitamin D’s regulatory role extends beyond bone and mineral homeostasis to include cardiovascular and metabolic functions, suggesting a cooperative mechanism with cardiotonic steroids within the body. Bufalin has been proposed as a valuable tool for exploring VDR regulation by signals originating from the cell membrane and for understanding the pathophysiological mechanisms that link VDR to cardiovascular dysfunction and cancer (Nakano et al., 2005; Amano et al., 2009). Pre-clinical evidence on bufalin suggests that bufalin, potentially in combination with 1α,25-dihydroxy vitamin D3 or its synthetic analogs, could have therapeutic value in the treatment of myeloid leukemia (Amano et al., 2009). The authors found that bufalin treatment stabilized the nuclear expression of VDR and enhanced the expression of the VDR-target gene Cyp24A1 and cathelicidin antimicrobial peptide. They also found that bufalin effectively enhanced VDR activity induced by 1α,25-dihydroxy vitamin D3 (Nakano et al., 2005) and also expanded the levels of nuclear VDR expression by inhibiting its degradation. However, this effect was not observed in other cell types, such as THP-1 and U937 cells, suggesting that the effect of bufalin on nuclear VDR expression might be cell-type dependent. In addition to bufalin modulating the interaction of VDR and its cofactors, its treatment inhibited the proliferation of HL60 cells and enhanced their differentiation when induced by 1α,25-dihydroxy vitamin D3 (Nakano et al., 2005; Amano et al., 2009).

Despite bufalin’s therapeutic potential, there are challenges associated with its development into a drug. One challenge is its toxicity to normal cells and low solubility in aqueous solutions adds to the challenge. Nevertheless, preclinical studies have shown promising results, and further studies are warranted to unlock the full therapeutic potential of Bufalin.

**3. Tanshinone IIA:** Is a natural compound that is isolated from the root of *Salvia miltiorrhiza*, also known as Danshen, a traditional Chinese medicinal herb. The roots of Danshen have been used for centuries for the treatment of cardiovascular and cerebrovascular diseases and several other conditions (Li et al., 2018). Tanshinone IIA belongs to a group of phytochemicals known as diterpenoids, which are organic compounds with a diverse array of chemical structures. Tanshinone IIA contains a quinone substructure in its chemical structure, which gives it unique pharmacological properties (Ansari et al., 2021). A wealth of literature provides thorough reviews on the extensive range of pharmacological effects tanshinone IIA exerts on various bodily systems. These effects encompass anti-inflammatory, anti-tumor, anti-oxidative, and antimicrobial activities (Ansari et al., 2021; Bi et al., 2021; Subedi and Gaire, 2021). Moreover, preclinical studies have demonstrated the ability of tanshinone IIA to deter platelet aggregation and enhance blood circulation, thereby suggesting its potential utility in managing cardiovascular diseases. Such an outcome has proven beneficial in mitigating myocardial injury and reducing short-term cardiovascular events. Specifically, this has been observed in individuals with non-ST elevation acute coronary syndrome who underwent percutaneous coronary intervention and were administered sodium tanshinone IIA sulfonate (Mao et al., 2021). In addition, tanshinone IIA has been shown to have neuroprotective effects, which could make it useful in the treatment of neurodegenerative diseases like Alzheimer’s disease (Subedi and Gaire, 2021).

Years of research have revealed that tanshinone IIA may ameliorate diabetes and its associated complications, such as diabetic kidney diseases (Ansari et al., 2021). A recent study offered mechanistic insight into this process, demonstrating that high glucose hindered cell proliferation and spurred the transition of renal tubular epithelial cells to mesenchymal cells in HK-2 samples. Remarkably, the introduction of tanshinone IIA was able to reverse this high glucose-induced transition. It accomplished this by elevating the levels of VDR protein while concurrently reducing the levels of β-catenin and GSK-3b proteins, suggesting a potential inverse relationship between VDR and the Wnt/β-catenin pathway. This hypothesis was further validated by the team using VDR-siRNA transfection, where they found out that when cells were exposed to high glucose, the β-catenin pathway. However, upon the restoration of VDR levels through the introduction of tanshinone IIA, the inhibitory effect of VDR on the pathway was recovered, and the pathway’s activation was suppressed (Zeng and Bao, 2021). These findings indicate that tanshinone IIA may modulate VDR expression levels either directly or indirectly, which in turn may help mitigate cardiometabolic diseases, such as diabetic kidney diseases. However, the challenges of developing tanshinone IIA into a drug include its poor solubility in water and low bioavailability, which make it difficult to deliver effectively to the body.

**4. Vitexin:** Popularly referred to as ‘Mujingsu’ in Chinese, is a natural flavonoid compound that is found in a wide variety of Chinese medicinal plants. It is particularly abundant in the leaves of the *Vitex negundo* plant, also known as the Five-leaved Chaste Tree, which is a traditional Chinese medicinal herb used in the treatment of inflammation, infection, and other conditions (Liang et al., 2023). Vitexin has a chemical structure characterized by a flavone substructure, which belongs to the flavonoid family. It has been found to have a wide range of pharmacological activities, including anti-inflammatory, antioxidant, anti-tumor, and neuroprotective effects (Liu et al., 2018c; Zhao et al., 2021; Chen et al., 2022). Studies have also shown that vitexin has potential therapeutic effects for metabolic conditions such as liver injury and diabetes (Akter et al., 2021; Noor et al., 2022).

Several compelling studies suggest a potential role for vitexin in the treatment of osteoporosis, a disease characterized by weakened bones. For instance, reports indicate that vitexin inhibits RANKL-induced osteoclast formation, a process associated with bone resorption, and impedes lipopolysaccharide-induced osteolysis, thus preserving bone integrity (Jiang et al., 2019). Furthermore, vitexin has shown the ability to promote the proliferation of osteoblasts and increase calcium deposition in osteoblast-like cells (Lin et al., 2020). Such evidence points to vitexin’s potential to restore bone microarchitecture, particularly in osteoporosis. This potential was further demonstrated by a recent study that reported that vitexin’s positive impact on osteoblast biochemical indicators such as VEGFA, ALP, and Runx2 in the ovariectomized rat model (Liu et al., 2023a). Additionally, the authors found that vitexin appears to foster angiogenesis, the formation of new blood vessels, an essential process for bone health and regeneration. This dual role in promoting angiogenesis and osteogenesis suggests that vitexin could facilitate a process known as angiogenesis-dependent osteogenesis, which is crucial for bone health (Liu et al., 2023a).

Notably, vitexin was found to increase the expression of VDR in the femur tissue of osteoporosis rats (Liu et al., 2023a). VDR is known to be a significant target for osteoporosis treatment, and this increase in expression suggests that vitexin might exert its bone-protective effects through VDR modulation. Further supporting this idea, the authors show that vitexin directly binds to VDR with good affinity (Liu et al., 2023a). The VDR/eNOS signaling pathway is another critical player in angiogenesis and osteogenesis regulation, and vitexin seems to operate at least in part through this pathway. By activating the VDR/eNOS signaling, vitexin may promote bone angiogenesis, which in turn supports osteogenesis, thereby offering a potential strategy for osteoporosis treatment (Liu et al., 2023a). Therefore, vitexin’s apparent interaction with the VDR/eNOS signaling pathway and the subsequent effects on angiogenesis-dependent osteogenesis further solidifies vitexin’s potential as a novel therapeutic strategy for osteoporosis.

**5. Asperuloside:** Is a natural iridoid glucoside compound that is found in several plant species, including *Asperula odorata*, *Galium odoratum*, and *Galium verum.* These plants are commonly used in TCM and European folk medicine for treating various conditions, including inflammation, pain, and infection (Manzione et al., 2020). Characterized by its iridoid glycoside substructure, asperuloside is also considered a member of *O*-glycosyl compounds, which are defined by the presence of an *O*-glycosidic bond. Asperuloside is highly water-soluble and has a weakly acidic nature (Cha et al., 2020). Like other bioactive glucosides, asperuloside has been found to have a range of pharmacological activities, including anti-inflammatory, antioxidant, anti-obesity, and laxative effects (Cha et al., 2020; Nakamura et al., 2020). Studies have also shown that asperuloside has potential therapeutic effects for conditions such as cancer (Qi et al., 2023).

VDR signaling pathway has a crucial role in several physiological activities, such as bone development, anti-inflammatory response, and anti-tumor activities (Shang and Sun, 2017). Notably, a decrease in both the serum vitamin D levels and the expression of VDR in the colon has been observed in patients with colitis or colorectal cancer (Chen and Jin, 2022; Ma et al., 2023). In a study using a mouse model of colitis-associated cancer, treatment with asperuloside was found to significantly enhance the expression of VDR in the colon, leading to marked reductions in colitis symptoms and a decrease in both the quantity and size of tumors (Lu et al., 2022). This highlights the potential of asperuloside as a promising therapeutic agent for colorectal cancer. In-depth *in-vitro* investigations revealed that asperuloside not only increases VDR expression but also reduces Smad3 mRNA levels, thereby suggesting its potential to inhibit the development of the epithelial-mesenchymal transition via VDR regulation (Lu et al., 2022). Intriguingly, asperuloside was observed to enhance VDR signaling and suppress Smad3 mRNA in NF-κB knockout cells, implying that asperuloside may inhibit Smad3 mRNA through VDR signaling activation, independent of NF-kB signaling. This conclusion aligns with the observed reduction in the transformation from the epithelial phenotype to the more motile mesenchymal phenotype, along with a decrease in epithelial-mesenchymal transition markers (Lu et al., 2022). These findings underscore the potential of asperuloside to prevent colitis-associated cancer by hindering the development of EMT, a pivotal process in the disease’s progression. This beneficial effect is achieved through the regulation of the VDR/Smad3 pathway, further underscoring asperuloside’s potential in the fight against colitis-associated cancer.

**6. Berberine:** Berberine is an isoquinoline alkaloid that is found in many plant species, including *Berberis aristata*, *Coptidis rhizoma*, and *Hydrastis canadensis.* These plants are commonly used in TCM and Ayurvedic medicine for treating various conditions, including infections, inflammation, and metabolic disorders such as diabetes and biological aging (Wang et al., 2019). Berberine, in particular, is a popular natural compound that has been studied extensively and found to have a wide range of pharmacological activities, including but not limited to anti-inflammatory, antioxidant, anti-hyperglycemia, and antitumor effects (Wang et al., 2017). Study reports that have surfaced have demonstrated that berberine possesses potential therapeutic benefits for conditions mainly including cardiovascular disease and metabolic disorders. This is attributed to berberine’s capability to interact with key metabolic molecules, specifically AMPK, SIRT1, LDLR, PCSK9, and PTP1B (Feng et al., 2019). Berberine has shown promising effects in the context of alleviating irritable bowel syndrome (IBS) (Hou et al., 2019). VDR plays a crucial role in the homeostasis of the mucosal barrier by maintaining the integrity of junction complexes and promoting the healing capacity of the colonic epithelium. This is evident from studies showing that VDR-deficient mice are more prone to mucosal injury induced by dextran sulfate sodium (Kong et al., 2008). Also, the treatment with 1α,25-dihydroxy vitamin D3, an active form of Vitamin D, enhances tight junctions in Caco-2 monolayers, a model of human intestinal cells, by increasing junction protein expression and transepithelial electric resistance (Kong et al., 2008). This vitamin D-dependent effect on junction integrity is vital, as reduced VDR expression has been shown to decrease junction proteins and intestinal transepithelial electric resistance.

In the context of IBS, particularly the diarrhea-predominant form (IBS-D), vitamin D deficiency may compromise the mucosal barrier, leading to increased susceptibility to mucosal damage and worsening symptoms. In clinical studies, IBS-D patients receiving vitamin D supplementation experienced significant improvements in symptom severity, quality of life, hospital anxiety and depression, and visceral sensitivity index scores compared to a placebo group (Khalighi Sik et al., 2020). In a rat model of IBS-D, berberine improved symptoms in a dose-dependent manner. Importantly, berberine effectively promoted the activity of the vitamin D receptor response element promoter, enhancing the expression of tight junction proteins in colonic epithelial cells (Huang et al., 2023). The upregulated proteins included occludin and zonula occludens-1, crucial components of the intestinal mucosal barrier. This suggests that berberine can enhance the intestinal mucosal barrier function in IBS-D, primarily through promoting VDR activity. Importantly, it was found that berberine primarily targets the N-terminal region of the VDR to regulate VDR activity (Huang et al., 2023). These findings show that berberine represents a novel and potentially effective approach for treating IBS-D via targeting VDR.

**7. Astragaloside:** This is a natural bioactive compound that is extracted from the root of *Astragalus membranaceus*, a traditional Chinese herb commonly used in herbal formulations for various medicinal purposes (Yu et al., 2022). Astragaloside (3-*O-β-D*-xylopyranosyl-*6-O-β-D*-glucopyranosyl-cycloastragenol) has a complex chemical structure consisting of a triterpenoid backbone with a cycloartane structure, which belongs to the saponin family. Astragaloside has been found to have a range of pharmacological activities, including antioxidant, anti-inflammatory, and immunomodulatory effects (Zhou et al., 2023). Some meta-analyses focused on clinical and preclinical studies also indicate that astragaloside may offer potential treatment benefits for conditions including hepatic fibrosis, heart failure, neurological diseases, diabetes, and cancer (Costa et al., 2019; Xiao et al., 2020; Zhou et al., 2020; Han et al., 2021; Li et al., 2023). Further evidence is mounting to suggest that astragaloside’s anti-cancer properties are largely delivered through mechanisms that promote apoptosis and autophagy while simultaneously impeding cell growth, invasion, movement, and spread. In addition, astragaloside appears to modulate the tumor microenvironment, influencing factors like new blood vessel formation and both innate and adaptive immune responses (Zhou et al., 2023).

The use of laboratory-adapted *Vdr* KO animal models and cardiovascular tissue-specific *Vdr* null mice has revealed key insights into the role of vitamin D signaling in the cardiovascular system. Notably, mice lacking *Vdr* or *Cyp27B1* exhibited elevated renin levels, leading to an increase in angiotensin II, which in turn resulted in hypertension and cardiac hypertrophy. Furthermore, aged normocalcemic *Vdr* null mice on a rescue diet developed a series of cardiovascular complications, including endothelial dysfunction, increased arterial stiffness, augmented aortic impedance, and compromised systolic and diastolic functions. These issues are believed to be mechanistically linked to the lack of *Vdr* signaling, which chronically reduces the bioavailability of nitric oxide (NO)—a potent vasodilator—through decreased expression of NO synthesizing enzyme (Christakos et al., 2016). In light of these findings, astragaloside has been found to counteract the effects of endothelin-1 (ET-1), which induces hypertrophy and apoptosis in cardiomyocytes. Specifically, the investigation revealed that astragaloside treatment led to a dose-dependent increase in VDR and CYP27B1 mRNA expression levels to improve cardiomyocyte injury. The evident link between astragaloside and the modulation of VDR signaling strengthens the case for the viability of VDR as a potential target for cardiovascular disease treatment (Yunzhi et al., 2017). This also emphasizes the need for further exploration into the mechanisms of astragaloside’s effects, as it shows great promise for the treatment of cardiovascular diseases.

**8. Parthenolide:** Is a natural compound that is found in many plants, including feverfew (*Tanacetum parthenium*), leaf vegetables, and herbs such as chamomile. Feverfew has been used for centuries in traditional medicine to treat various conditions, including migraines, arthritis, and menstrual pain (Pareek et al., 2011). The chemical structure of parthenolide contains a lactone moiety and is classified as a germacrene sesquiterpene lactone. Parthenolide has been found to have a range of pharmacological activities, including anti-inflammatory, anticancer, redox-modulation, and neuroprotective effects (Freund et al., 2020). Studies have also shown that parthenolide has potential therapeutic effects for conditions such as leukemia, Alzheimer’s disease, and migraines (Materazzi et al., 2013; Yang et al., 2017; Fan et al., 2023). In addition to these diseases, parthenolide has promising effects on diabetes and its related complications (Liu et al., 2023b). Mounting evidence also has established a close relationship between VDR signaling and diabetes, as well as diabetic nephropathy and insulin resistance. For example, high serum levels of 1α,25-dihydroxy vitamin D3 decrease the risk for diabetic nephropathy (Chen et al., 2023). Besides, increasing VDR levels may alleviate proteinuria, renal fibrosis and inflammation and prevent podocyte damage (Zhang et al., 2010; Wang et al., 2012).

Treatment with parthenolide resulted in improvements in glomerular mesangial expansion, glomerular hypertrophy, and albuminuria. In addition, parthenolide effectively ameliorated diabetic nephropathy-associated complications, including renal tubular basement membrane thickening, renal fibrosis, proteinuria excretion, and high blood sugar levels. In a cellular context, parthenolide significantly improved high glucose-induced apoptosis and inflammation in MPC5 cells, a podocyte cell line. It was also observed to increase the expression levels of matrix-related proteins such as nephrin and podocin, key elements in maintaining podocyte integrity (Yan et al., 2022).

Parthenolide has shown potential as a DNA methylation inhibitor—it was observed to inhibit DNA methyltransferase 1 (DNMT1) (Liu et al., 2009), a noteworthy effect considering DNMT1 was found to be upregulated in diabetic nephropathy mice and high glucose-induced MPC5 cells (Yan et al., 2022). This upregulation contributes to the hypermethylation of the VDR gene, which is significantly associated with lower VDR gene expression levels and vitamin D levels (Guan et al., 2022). Consequently, in high glucose-induced podocytes, VDR protein expression was significantly reduced, while the methylation level of its promoter was increased. Parthenolide demonstrated its ability to promote VDR protein expression while inhibiting the methylation level of the VDR promoter. Parthenolide’s promotion of VDR expression led to the upregulation of the AKT phosphorylation level in HG-induced podocytes, a process that was reversed by the addition of the AKT inhibitor MK2206(193). The multifaceted capabilities of parthenolide, from its influence on diabetic nephropathy-associated complications to its modulation of VDR expression, position it as a potential therapeutic agent in managing diabetic nephropathy.

## 6 Challenges, limitations, and future directions

The VDR plays a leading role in many physiological processes in various biological systems. As an intracellular receptor for vitamin D, VDR helps regulate the genomic effects essential for maintaining general wellbeing, pointing to another reason why avoiding vitamin D deficiency is valuable to overall health. In addition, its wide distribution in different cells and tissues explains the numerous therapeutic functions of VDR modulation in different systems, subsequently implicating its alteration in different body system diseases. This also highlights that more VDR-dependent genes may be discovered in the future. These genes could provide insights into and solidify our understanding of how VDR contributes to overall health or causes VDR-dependent diseases—a spectrum of conditions that are either directly influenced by the activity of the VDR protein or indirectly affected through its regulatory impact on various physiological processes.

To date, there is insufficient evidence to support whether using vitamin D or its synthetic analogs can help treat common but detrimental non-communicable diseases such as cancers, diabetes, heart and vascular diseases, and many more. However, numerous observational clinical studies have established the relationship between vitamin D supplementation and these non-communicable diseases, hinting that there is a need to use appropriate disease models to delve into how VDR activity can be modulated to help treat these diseases. New advances in methods and bioinformatic tools are also providing a better understanding of the structural interactions between VDR and its protein partners, which will help explain the selective effects of VDR in different target tissues and how the expression of VDR target proteins can be controlled. While most known VDR modulators are steroids, nonsteroidal modulators have also been identified with strong therapeutic effects without causing high calcium levels in the blood.

TCM have been historically recognized for their multi-target, multi-component therapeutic effects. Interestingly, recent investigations have highlighted the potential of various TCM-derived bioactive compounds in modulating VDR activities, signifying a promising avenue for therapeutic applications in VDR-dependent diseases. Nevertheless, the use of TCM in VDR modulation presents its own set of challenges and limitations. One significant challenge lies in the complexity of TCM, given that each preparation typically comprises multiple bioactive compounds, each of which may interact differently with the VDR. Therefore, identifying the specific component responsible for the therapeutic effect could be laborious and complex. Additionally, there are limitations concerning the quality control of TCM preparations, the quantification of their active ingredients, and the standardization of treatment protocols. The interindividual variability in response to TCM therapies also presents a significant challenge. These differences could be attributed to genetic factors, diet, gut microbiota, and other environmental influences that can significantly affect the absorption, distribution, metabolism, and excretion of TCM compounds.

Moreover, the underlying mechanisms of how TCM modulates VDR are still largely unexplored, which limits our understanding on the therapeutic outcomes. A better understanding of these mechanisms will allow the development of more effective therapeutic strategies for VDR-dependent diseases. On the bright side, the integration of modern scientific techniques with traditional medical knowledge offers promising future directions. For example, utilizing advanced bioinformatics and molecular biology tools has helped elucidate the specific interactions between TCM compounds and VDR. These approaches have also aided in the identification and characterization of new TCM-based VDR modulators, enabling the design of more effective therapeutic strategies. This suggests that in the coming years, the drug discovery field of VDR research will continue to grow and provide new insights into novel TCM-based nonsteroidal modulators of VDR that will help us better appreciate the implications of VDR signaling in health and disease. However, to better have this sufficient evidence, studies must push beyond the primary *in-vitro* activity assays to more complex structural-activity study designs that seek to offer atomic-level knowledge about the dynamics between VDR and its activity-supporting partners and these TCM compounds. Such future advances may provide a rationale understanding for addressing most of the mechanistic issues associated with how the genomic effects of VDR are stimulated in the presence of TCM interventions.
